# Blood Lead and Water Treatment

**DOI:** 10.1289/ehp.10453

**Published:** 2007-10

**Authors:** June M. Weintraub

**Affiliations:** San Francisco Department of Public Health/Environmental Health Section, San Francisco, California, E-mail: june.weintraub@sfdph.org

Miranda et al. (2007) recently reported the results of their investigation into the relationship between blood lead levels and residual water treatment in two locations in North Carolina. Their conclusion that “the change to chloramine disinfection may lead to an increase in blood lead levels, the impact of which is progressively mitigated in newer housing” is not borne out by their analysis. Their ecologic study design cannot be used to determine causation, and their recommendation to change lead screening strategies based on their study seems premature.

Their analysis relied on ecologic assignment of drinking water exposures based on residence location, census-level exposures for housing age, and other risk factors for lead exposure. They provided no information on differences in water chemistry, such as pH or corrosivity, or on the presence of lead in the distribution systems or service lines. These characteristics are important to determine the likelihood of lead leaching, regardless of the type of residual disinfectant used. In fact, the water serving Wayne County, North Carolina, is heterogeneous. Whereas the city of Goldsboro is served by surface water sources, the rest of Wayne County is served by seven smaller sanitary districts that rely on groundwater. Further, among the groundwater sources there is considerable variability in the water quality. The Wayne Water Districts, erroneously referred to by Miranda et al. (2007) as “Wayne Water Systems,” are composed of five of the seven sanitary districts in Wayne County and had 39 wells in 2006, about half of which receive no treatment. The remaining half are treated for iron removal, fluoride, chlorine, and phosphate. The pH for Wayne Water Districts water ranges from 6.5 to 7.5, whereas Goldsboro Water usually maintains a pH > 8.0.

Miranda et al. (2007) stated that they

expected that the effect of chloramines on [blood lead levels] would be less important and eventually unimportant as [they] moved into newer and newer housing stock.

However, they offered no explanation for their *a priori* hypothesis of interaction. In fact, according to the U.S. Environmental Protection Agency (1993), the opposite would be true:

Lead levels decrease as a building ages. This is because, as time passes, mineral deposits form a coating on the inside of the pipes (if the water is not corrosive). This coating insulates the water from the solder.

In the categorical analysis, stratifying housing age by 25-year categories may have led to misclassification. In 1988 the Consumer Product Safety Commission (CPSC 1988) began enforcing the Federal Hazardous Substances Act restricting the use of lead solder in plumbing. The cutoffs used by Miranda et al. (2007) combine the years 1976–1988 together with newer housing stock that would not have lead solder in the plumbing. It is also curious that the tax parcel data from which they assigned housing age characteristics differs markedly from that reported in the 2000 U.S. Census (U.S. Census Bureau 2000). Where Miranda et al. (2007) reported 15.6% of Wayne County housing stock built before 1925, the U.S. Census reported just 7.6% built before 1939 (U.S. Census Bureau 2000).

Miranda et al. (2007) were unable to include 25% of records; missing these data could have biased the results. For example, if a higher proportion of the missing children were from Seymour Johnson Air Force Base, which is annexed to the city of Goldsboro and which receives water from the Goldsboro Water System, this could have overestimated the effect of switching to chloramine if the missing children had lower blood lead levels. Children who reside on military bases tend to have lower lead exposures, regardless of the housing stock (Stroop et al. 2002).

Miranda et al. (2007) should have considered the possible impact of Hurricane Floyd. The September 1999 hurricane left many homeless; flooding, demolition, and construction activities could well have affected children’s blood lead levels to a greater extent in Goldsboro compared with the rest of Wayne County. Indeed, the raw blood lead level data show an increase county-wide in the year 2000, supporting an effect of the hurricane.

The analysis conducted by Miranda et al. (2007) is interesting, and additional studies with individual-level exposure measurements should be conducted. However, their study does not provide a basis for recommending a broad alteration of blood lead screening strategies. States need to do better to ensure that blood lead screening strategies are inclusive, especially for low-income children who are at the greatest risk of undetected elevations in blood lead levels. The recommendation of Miranda et al. (2007) to exclude certain children based on water-disinfection practices could have substantial unintended impacts. In accordance with recommendations of the U.S. Preventive Services Task Force (2007), a more prudent approach to prevent lead exposure via drinking water is that municipalities ensure careful corrosion control and remove lead service lines and distribution pipes, regardless of the method of residual disinfectant used.
